# Water quality assessment using IRWQI_sc_ and NSFWQI water quality indicators; A case study: Talar River (Iran)

**DOI:** 10.1016/j.heliyon.2025.e41812

**Published:** 2025-01-10

**Authors:** Mohammad Roshani-Sefidkouhi, Fatemeh Mortezazadeh, Masoumeh Eslamifar, Esmaeil Babanezhad, Masoomeh Sheikhi, Fathollah Gholami-Borujeni

**Affiliations:** a Department of Environmental Health Engineering, Student Research Commitee, Faculty of Health, Mazandaran University of Medical Sciences, Sari, Iran; b Department of Environmental Health Engineering, School of Public Health, Tehran University of Medical Sciences, Tehran, Iran; c Center for Solid Waste Research (CSWR), Institute for Environmental Research (IER), Tehran University of Medical Sciences, Tehran, Iran; d Faculty of Health, Mazandaran University of Medical Sciences, Sari, Iran; e Department of Environmental Health Engineering, Faculty of Health, Mazandaran University of Medical Sciences, Sari, Iran; f Faculty of Health, Mazandaran University of Medical Sciences, Sari, Iran; g Department of Environmental Health Engineering, Health Sciences Research Center, Mazandaran University of Medical Sciences, Sari, Iran

**Keywords:** Water quality, Talar River, IRWQI_SC_, NSFWQI, Mazandaran, Iran

## Abstract

One of the driest countries in the world, Iran has been facing several challenges related to water shortage, leading to serious social, economic, and environmental impacts. Given the escalating urbanization and industrial development within Iran, a comprehensive assessment of surface water quality utilizing both the US National Sanitation Foundation Water Quality Index (NSFWQI) and the Iran Water Quality Index for surface water (IRWQI_sc_) is imperative. This study aimed to evaluate the water quality of the Talar River in Mazandaran, Iran, in 2023, through the IRWQI_sc_ and NSFWQI. This study was conducted in the Talar River, located in Mazandaran Province in Iran. Water samples from the Talar River were collected to assess 11 physicochemical parameters from 10 sampling Points. A total of 60 samples were collected monthly during winter and summer 2023. The IRWQI_sc_ and NSFWQI were selected to assess the overall water quality of the Talar River. Nitrate (NO_3_^−^) concentrations ranged from 29.32 to 85.89 mg/L across seasons, with average values falling within World Health Organization (WHO) standards. Phosphate (PO_4_^3−^) and Chemical Oxygen Demand (COD) levels exceeded WHO limits on occasion, while turbidity and electrical conductivity (EC) consistently surpassed both WHO and Environmental Protection Agency (EPA) guidelines. pH and Biochemical Oxygen Demand (BOD) levels remained within acceptable ranges. IRWQI_sc_ and NSFWQI indicated generally poor water quality across all sampling sites, with slight seasonal variations suggesting slightly better conditions in summer for IRWQI_sc_ and winter for NSFWQI. This study indicates that NO_3_^−^ and PO_4_^3−^ levels meet WHO limits, but COD, turbidity, and EC exceed the recommended threshold. The IRWQI_sc_ and NSFWQI highlight poor conditions resulting from agricultural and industrial pollution. Urgent integrated management is essential to mitigate these impacts and safeguard river health in the face of environmental pressures.

## 1 Introduction

Water is an indispensable resource for all living organisms, including humans, and plays an important role in national development [1]. On the other hand, Water pollution poses a significant global challenge, particularly in developing countries such as Iran [2]. During recent decades, there has been an evident decline in water quality, attributed to the increasing growth in population, industrialization, and different types of pollutants coming from urban and industrial or agricultural sources [3]. A straightforward approach to assessing water quality involves the utilization of a water quality meter [4]. Traditionally, water quality assessments relied on comparisons to established standards. While this approach offers simplicity, it may not provide a comprehensive and precise evaluation of water quality, particularly for managers and decision-makers who necessitate detailed information regarding high-quality water resources. To overcome this limitation and to improve the assessment of water quality characteristics, the Water Quality Index (WQI) was developed. The WQI employs a numerical formula to transform water quality attributes into quantitative scores, thereby providing a representation of the overall water quality condition [[5], [6], [7]]. These indices are simple but powerful tools for water quality assessment, using mathematical models representing a range of water quality conditions, from poor to excellent [8]. The classification of water quality using WQI allows researchers to assess the impact caused by the contaminants. Eventually, this may provide politicians and decision-makers with the potential to take proper action for water resource management and to choose appropriate sources according to the intended use of water [9]. In the last two decades, several water quality indices have been proposed all over the world. Amongst various indices, the National Sanitation Foundation Water Quality Index (NSFWQI) is considered the best index due to its relatively higher accuracy, simplicity, wide applicability, and easy availability of the required parameters [[10], [11], [12]]. In contrast, the Iran Water Quality Index for surface water (IRWQI_sc_) was developed to deal with the major issues concerned with water quality that have been generally faced in Iran [9]. The calculation of the IRWQI_sc_ refers to developing some indicators influenced by different natural situations and water quality problems in Iran. It is concerned with creating a wide vision of water quality in the country by taking on board the challenges involved [13]. Given the importance of this issue, numerous studies have been conducted to evaluate the quality of water resources. A study conducted evaluated the water quality status of the Zarin-Gol River using both the NSFWQI and IRWQI_sc_. Both indices yielded moderate water quality classifications, with NSFWQI values ranging from 50 to 70 and IRWQI_sc_ values ranging from 45 to 70 [14]. Conversely, Mahrouyan et al. evaluated the Shahrud River's water quality using the NSFWQI. The results revealed good water quality at station 1 during summer, while other stations exhibited moderate quality. In winter, stations 1 and 4 displayed good water quality, whereas the remaining stations demonstrated moderate water quality. Overall, the Shahrud River's water quality was classified as good to average [15]. A study employed NSFWQI and Geographic Information System (GIS) to evaluate the spatial distribution of water quality in the Pir-e Ghar River. The results revealed moderate to good water quality conditions at all stations, with the highest NSFWQI value of 78 observed at the first station and the lowest value of 66 at the fifth station. The increasing pollution load downstream of the Sarab station led to a deterioration in water quality [16]. The other study evaluated the water quality of the Karaj and Kan Rivers using the NSFWQI, IRWQI_sc_, and WQI. The NSFWQI classified water quality as ranging from bad to moderate, the IRWQI_sc_ as ranging from very bad to relatively good, and the WQI as good. Despite these inconsistencies, the overall assessment indicated that the water in both rivers was suitable for drinking and agricultural purposes [17]. According to the Fathi et al.'s evaluation of Choghakhor Lagoon's water quality using the WQI revealed a very poor water quality classification, rendering it unfit for human consumption [18]. Khalifa et al. employed IRWQI_sc_ to evaluate water quality at 16 stations along the Zarineh Rood River across four seasons. The results revealed that none of the stations fell within the very good or very bad categories, with only station 16 in the spring being classified as bad [19]. Aghaee et al. assessed the water quality of the Chil Chai River using the Index of IRWQI_sc_. Their findings indicated a relatively good water quality status in the upstream reaches, which gradually deteriorated downstream. While the water was deemed suitable for agricultural applications, it did not meet the prescribed standards for potable water consumption [20]. Similar studies have been carried out in other rivers in Asia [21,22], as well as in Europe and Africa [[23], [24], [25]]. Despite the increasing level of international concern over water quality in river systems, the specific challenges of water resource management in Iran, especially regarding water pollution, have not been thoroughly studied. Previous research on water quality indices, such as IRWQI_sc_ and NSFWQI, has predominantly been conducted in disparate geographic contexts, with limited application to complex river systems like the Talar River. This research gap is especially pronounced in regions like Mazandaran, where a confluence of natural and anthropogenic factors contributes to diverse water quality issues. The Talar River, a vital water resource for local communities, agriculture, and industry, is subjected to significant pollution from both point and non-point sources, including agricultural runoff, industrial effluent, and urban wastewater [26]. To address this research gap, this study integrates the IRWQI_sc_ and NSFWQI indices with a comprehensive spatial and temporal sampling design to provide a detailed, localized assessment of water quality across the entire river system. The primary objective of this research is to evaluate the water quality of the Talar River (Mazandaran, Iran) in 2023 using the IRWQI_sc_ and NSFWQI indices. This study aims to provide precise information about the water quality status from the river's source to its mouth, leveraging the aforementioned indicators.

## 2 Materials and methods

### 2.1 Study area

**Fig. 1:**
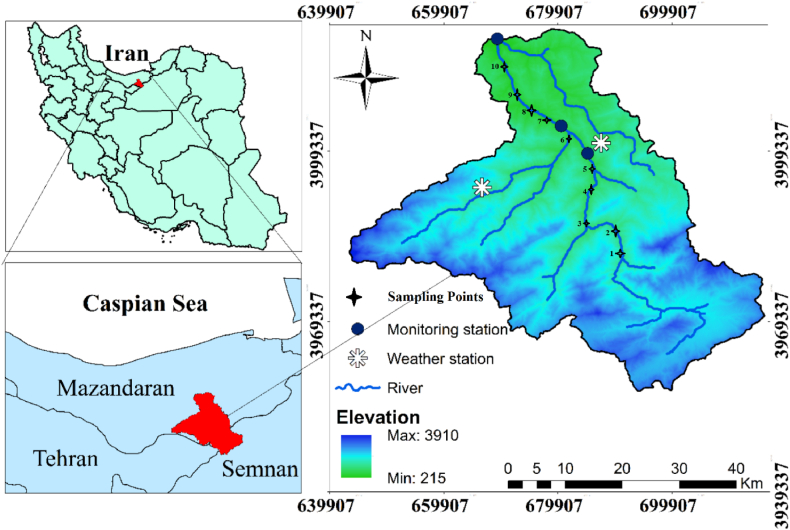
Study area and sampling points of Talar River.

This study was conducted on the Talar River, situated in Mazandaran Province, Iran (36°00′N to 32°11′N latitude, 66°36′E to 63°23′E longitude). Originating at an altitude of 3000 m in the Alborz Mountain range, specifically from the Veresk and Khatirkuh peaks, the river traverses the western region of Qaemshahr after merging with several tributaries in Savadkuh. Subsequently, it flows for approximately 120 km through Simorgh City before culminating in the Caspian Sea. The majority of the river's course winds through the mountainous and forested landscapes of Savadkuh City, encompassing the areas of Pol-e Sefid, Zirab, and Shirgah [27]. Major environmental challenges face the Talar River, mainly sourced by mining sand and gravel along Khatirkouh Road, and raise the turbidity and sedimentation in the river. Moreover, downstream is the Kiakla landfill, with its leachate directly discharged into the river, polluting it seriously. Moreover, the recent boom in tourism, construction, and agricultural and industrial activities has enhanced effluent discharge and augmented the severity of the pollution [28]. Additional characteristics of the Talar River, along with the geographical distribution of the sampling stations, are presented in Table 1 and Fig. 1, respectively.

**Table 1:** Characteristics of the Talar River (according to Hosseini et al. (2011) [29]).

NO.	Characteristic	Amount
1	Area of the drainage basin	2902.28 km^2^
2	Average annual precipitation	33.72 m^3^/s (Shirgah Station)
3	Length of the main branch of the river	160 km
4	Mean height	1690 m
5	Average slope of the field	22.54 %
6	Flow direction	Southeast to Northwest
7	Number of hydrometric stations	11 active stations

### 2.2 Water sampling and analysis

This sampling framework involved fixed and variable stations along the course of the river. Based on this framework, 10 encompassing sets of sampling stations were developed whose geographical positions and attributes are shown in Fig. 1. The criteria for the selection of these stations was an extensive review of the area, pointing out potential sources of pollution, mostly agricultural runoffs, and Khatirkuh sand factories. A detailed study of the course of the river on 1:50,000 scale maps had been done to decide on the exact sampling site within each station. This was particularly to assess entry points of pollutants and optimum locations that could give representative samples. A total of 60 water samples were collected monthly during both the rainy and dry seasons of 2023. At each station, three samples were obtained from distinct locations along the river's cross-section. Composite sampling was employed along the river. For microbial analysis, sterile glass bottles were employed to preserve sample integrity. Polyethylene bottles were utilized for chemical analyses due to their durability and chemical resistance. Upon collection, samples were immediately refrigerated at 4 °C or lower to inhibit microbial growth and chemical degradation. Robust packaging methods were employed to guarantee the integrity of the samples during transportation. Standard methods for water and wastewater analysis were employed to measure the concentrations of nitrate (NO_3_^−^), ammonium (NH_4_^−^), phosphate (PO_4_³^-^), biochemical oxygen demand (BOD), chemical oxygen demand (COD), total hardness (TH), and fecal coliform (FC) [30]. On the other hand, pH, temperature, dissolved oxygen (DO), electrical conductivity (EC), and turbidity were measured using a CYBERSCAN pH 5500 device, a DO meter-8403 AZ device, an AQUA LYTIC CON200 EC Meter, and a HACH 2100P portable instrument, respectively. All instruments were calibrated in the laboratory before field measurements.

### 2.3 Water quality indices

The IRWQI_sc_ and NSFWQI were selected as the primary indices to assess the overall water quality of the Talar River and to facilitate comparative analysis of their performance. A brief overview of these indices is provided below.

#### 2.3.1 IRWQI_sc_

The IRWQI_sc_, a widely utilized index for assessing surface water quality, was developed specifically to address Iran's unique natural conditions and resource-related challenges. Eleven water quality parameters (Table 2) are incorporated into the IRWQI_sc_ calculation (Equation (1)). According to the Iranian Guideline for Calculating Water Quality Index, even if fewer than eleven parameters are measured, Equation (1) should be applied without modification [9].

(1) $$\text{IR}{\text{WQI}}_{\text{SC}}={\left[\sum_{i=1}^{n}{I_{i}}^{W_{i}}\right]}^{\frac{1}{\gamma }}$$

(2) $$\gamma =\sum_{i=1}^{n}W_{i}$$

(3) $$0\leq I_{i}\leq 100$$

where Iᵢ and Wᵢ represent the sub-index value and weight coefficient of parameter i, respectively; γ is the sum of the weights; and n is the total number of water quality parameters. The sub-index values were determined using the sub-index rating curves of the IRWQI_sc_ provided by the Department of Environment (DOE) of Iran. The classification of index scores for water quality grading is presented in Table 2 [9]. Equation (2) calculates the sum of the weights given to all water quality parameters in IRWQI_sc_. Wᵢ represents the weight assigned to the i-th water quality parameter. This weight reflects the relative importance of that parameter in determining the overall water quality [9,31].

**Table 2:** Water quality parameters and their weights according to NSFWQI and IRWQI_sc_.

NSFWQI		IRWQI_sc_	
Parameter	weight	Parameter	weight
DO_sat_	0.17	DO_sat_	0.097
pH	0.11	pH	0.051
Turbidity	0.08	Turbidity	0.062
NO_3_^−^	0.1	NO_3_^−^	0.108
PO_4_^3-^	0.1	PO_4_^3-^	0.087
FC	0.16	FC	0.14
BOD	0.11	BOD	0.117
TS	0.07	NH_4_^+^	0.090
Temperature	0.1	COD	0.093
		EC	0.096
		TH	0.059

Also, γ, is used in the main IRWQI_sc_ equation (Equation (1)) as the exponent for the summation term. This exponent ensures that the influence of each parameter on the final index value is proportional to its assigned weight. On the other hand, Equation (3) defines the range of values for the sub-index (Iᵢ) of each water quality parameter. The sub-index (Iᵢ) indicates the water quality status of a parameter, ranging from 0 to 100, with 0 being the worst and 100 being the best [9].

#### 2.3.2 NSFWQI

NSFWQI is one of the oldest and most widely used quantitative indicators of water quality [32,33]. The calculation of the NSFWQI, as outlined in Equation (4), involves two key factors: the weight and quality of each parameter. By assigning values to each parameter, an index value is calculated for each, and the final NSFWQI is determined by averaging these individual index values [34]. Table 2 presents the 9 parameters included in the NSFWQI and their corresponding weight coefficients.

(4) $$\text{NSFWQI}=\sum_{i=1}^{n}Q_{i}W_{i}$$

**Fig. 2:**
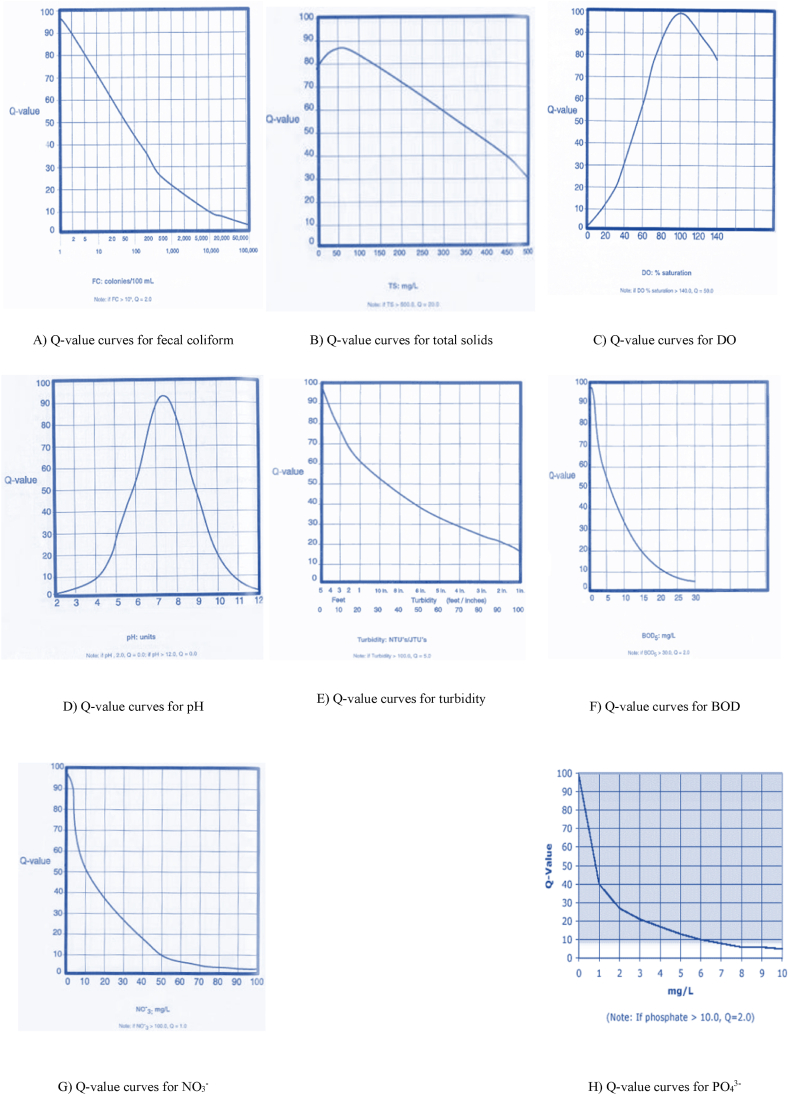
Index curves (A–H) to determine the quality parameters (Q) in the NSFWQI model [32].

To obtain quality parameter (Q), index curves are used, and these curves for the various parameters are shown in Fig. 2 [A-H]. It should be noted that if the number of FC colonies is greater than 100,000, the quality index equals 2; if total solids (TS) are greater than 500 ppm, the quality index equals 20; if DO is greater than 140 percent, the quality index equals 50; if pH is greater than 12 or less than 2, the quality index equals 0; if turbidity is greater than 100 NTU, the quality index equals 5; if BOD is greater than 30 ppm, the quality index equals 2; if NO_3_^−^ is greater than 100 ppm, the quality index equals 1; and if PO_4_^3−^ is greater than 10 ppm, the quality index equals 2 [35]. Fig. 2 [A-H] show how to calculate the quality index for the parameters. Water quality grades were assigned based on index scores calculated for stations, using pre-established classification (Table 3).

**Table 3:** Water quality classification according to NSFWQI and IRWQI_sc_.

NSFWQI		IRWQI_sc_	
Score	Classification	Score	Classification
0–24	Very bad	Less than 15	Very bad
25–49	bad	15–29.9	bad
50–69	Medium	30–44.9	Relatively bad
70–89	Good	45–55	Moderate
90–100	Excellent	55.1–70	Relatively good
		70.1–85	Good
		More than 85	Very good

## 3 Results

### 3.1 Descriptive statistics of seasonal variations in water quality parameters

**Fig. 3:**
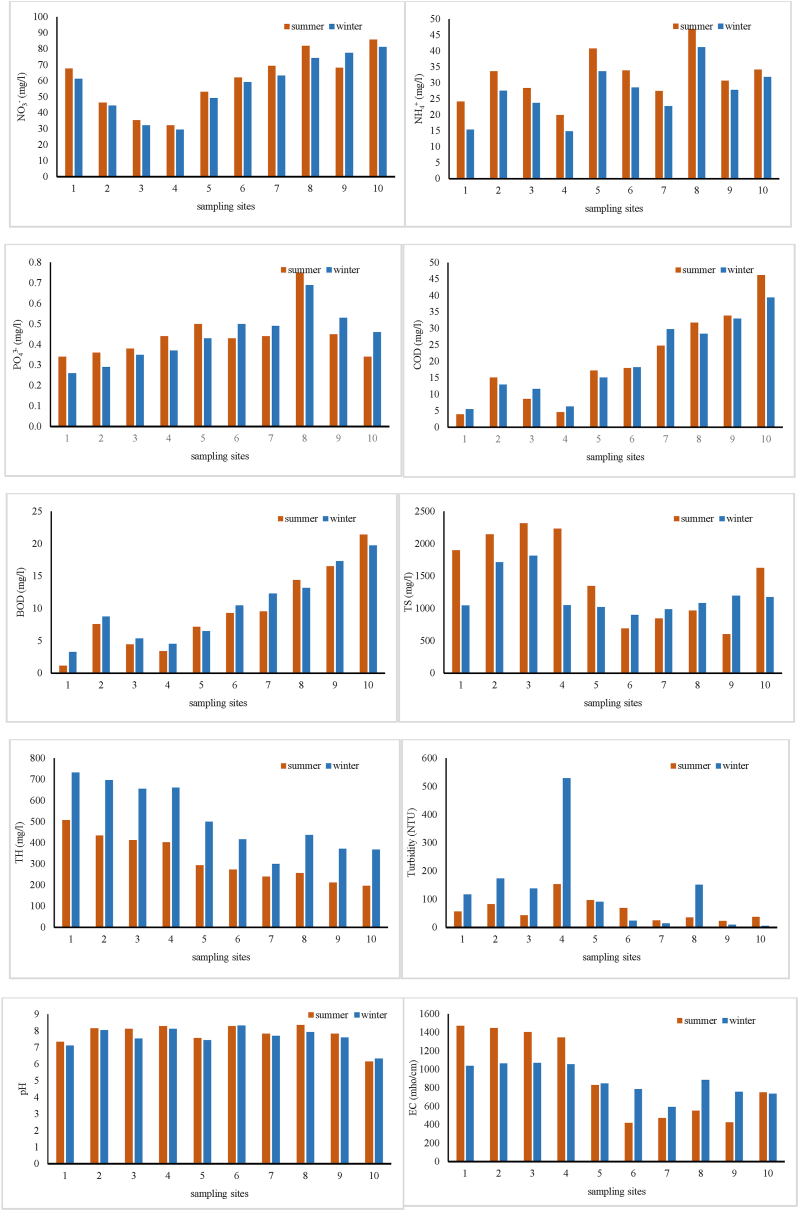

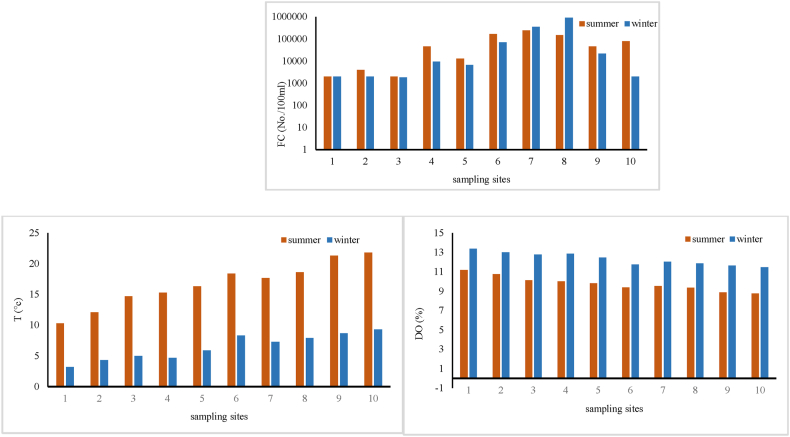
Variation of water quality parameters in different sampling sites of Talar River.

Table 4 presents a statistical summary of the Talar River's water quality parameters, including minimum, maximum, median, mean ± standard deviation (SD), and Relative Standard Deviations (RSD). As indicated in Table 5, NO_3_^−^ concentrations ranged from 29.32 to 81.23 mg/L in winter and 32.14–85.89 mg/L in summer. While the mean ± SD values for both seasons were comparable, the maximum PO_4_³^-^ concentrations exceeded the WHO standard of 0.5 mg/L. However, the average PO_4_³^-^ levels in both seasons remained within the acceptable WHO range. Conversely, the average COD in both seasons (19.99 and 20.4 mg/L) surpassed the standards set by both the WHO and EPA. Similarly, the average turbidity values (125.2 and 62 NTU) in both seasons exceeded the limits established by both organizations. The EC values were also higher than the limits set by the WHO (below 400 μS/cm) and EPA (200–800 μS/cm) in both seasons. Nevertheless, the pH values in both seasons remained within the acceptable range according to WHO and EPA standards. Additionally, the average BOD in both seasons (10.13 and 9.48 mg/L) was lower than the WHO and EPA standards. Fig. 3 visually represents the seasonal variation of water quality parameters across different sampling sites within the Talar River.

**Table 4:** Statistical summary of water quality parameters in Talar River [33,34].

Season	Winter					Summer					WHO	EPA
Parameters	Min	Max	Median	Mean ± SD	RSD	Min	Max	Median	Mean ± SD	RSD		
NO_3_^−^, mg/L	29.32	81.23	60.33	57.23 ± 18.14	0.32	32.14	85.89	64.92	60.21 ± 18.2	0.30	45	50
NH_4_^+^, mg/L	14.83	41.12	27.63	26.70 ± 8.04	0.30	19.92	46.84	32.12	31.97 ± 7.81	0.24	–	advisory level of 30 mg/L
PO_4_^3−^, mg/L	0.26	0.69	0.45	0.43 ± 0.12	0.29	0.34	0.75	0.44	0.44 ± 0.12	0.27	0.5	0.01–0.03
COD, mg/L	5.49	39.35	16.60	19.99 ± 11.80	0.59	3.89	46.19	17.60	20.4 ± 13.75	0.67	<3	5
BOD_5_, mg/L	3.27	19.73	9.60	10.13 ± 5.51	0.54	1.14	21.41	8.43	9.48 ± 6.29	0.66	5	3
TS, mg/L	899.80	1812.20	1066.05	1198.45 ± 309.998	0.26	605	2316	1485.50	1466.62 ± 664.152	0.45	500 mg/L	500 mg/L
TH, mgCaCO_3_/L	300	732	468	513.6 ± 158.21	0.31	197	507	283	322.5 ± 107.22	0.33	10–500	151–250
Turbidity, NTU	5	529	104	125.2 ± 155.64	1.24	23	153	49.50	62 ± 40.10	0.65	5	5
pH	6.33	8.32	7.65	7.61 ± 0.57	0.08	6.15	8.36	7.99	7.79 ± 0.66	0.09	6.5–8.5	6.5–9
EC, μS/cm	591	1071	867	883.3 ± 168.42	0.19	419	1472	790	911.7 ± 455.62	0.50	below 400 μS/cm	200 to 800 μS/cm
T,°C	3.20	9.30	6.60	6.46 ± 2.11	0.33	10.30	21.80	17	16.65 ± 3.69	0.22	26.3–32.3	typically, below 20 °C for taste.
DO, %	11.48	13.39	12.26	12.33 ± 0.65	0.05	8.78	11.21	9.67	9.78 ± 0.77	0.08	Minimum 5 mg/L	Minimum 5 mg/L
FC, No./100 ml	1800	920000	8050	138590 ± 294837.07	2.13	2000	240000	46000	75200 ± 83742.72	1.11	0 per 100 ml	0 per 100 ml

**Table 5:** Statistical summary of IRWQI_sc_ and NSFWQI in Talar River.

Index	Winter					Summer				
	Min	Max	Median	Mean ± SD	RSD	Min	Max	Median	Mean ± SD	RSD
IRWQI_sc_	12.30	20.90	18.30	17.86 ± 2.72	0.15	13.90	21.60	17.90	18.02 ± 2.49	0.13
NSFWQI	27.70	41.14	35.95	35.53 ± 3.94	0.11	23.59	40.40	30.05	30.88 ± 5.14	0.16

### 3.2 Water quality assessment using IRWQI_sc_ and NSFWQI

**Fig. 4:**
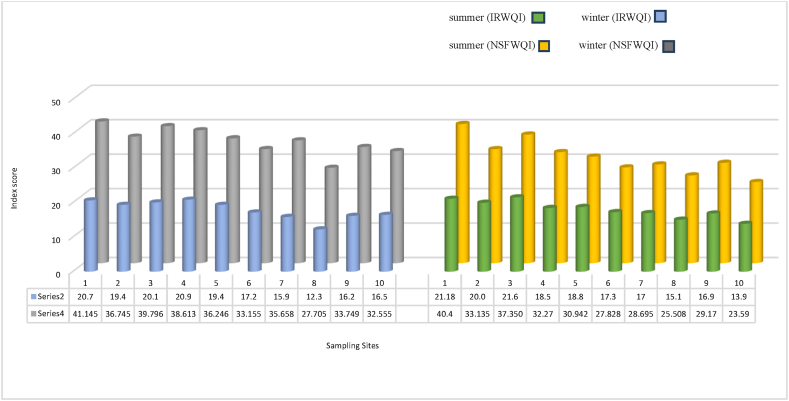
IRWQI_sc_ and NSFWQI in different sampling sites of Talar River.

Table 5 presents a statistical summary of two water quality indices, the IRWQI_sc_ and NSFWQI, for the Talar River during the winter and summer seasons. The indices were evaluated based on minimum, maximum, median, mean ± standard deviation (SD), and relative standard deviation (RSD) values. The relatively high standard deviations observed for both indices indicate substantial variability in water quality within each season. The proximity of the median values to the mean values suggests a relatively symmetric distribution of the data. Both indices exhibited similar seasonal trends. While the IRWQI_sc_ indicated slightly better water quality during the summer season, the NSFWQI suggested improved water quality during the winter season. Fig. 4 presents a comparative analysis of the IRWQI_sc_ and NSFWQI across various sampling sites along the Talar River during the summer and winter seasons. Fig. 4 employs a column chart to visualize the water quality indicators at each site. The x-axis represents the spatial distribution of sampling sites along the river, while the y-axis indicates the corresponding index values.

Table 6 provides an overview of the average IRWQI_sc_ and NSFWQI scores, along with their corresponding status classifications, for each sampling site during the winter and summer seasons. In winter, the IRWQI_sc_ scores ranged from 12.3 to 20.7, with an average score of 17.86. Similarly, the NSFWQI scores ranged from 27.7 to 41.14, with an average score of 35.53. During the summer season, the IRWQI_sc_ scores ranged from 13.9 to 21.6, with an average score of 18.02, while the NSFWQI scores ranged from 23.59 to 40.4, with an average score of 30.88.

**Table 6:** Seasonal score and status of IRWQI_sc_ and NSFWQI and average status.

Sampling site	Winter				Summer			
	IRWQI_sc_		NSFWQI		IRWQI_sc_		NSFWQI	
	Score	Status	Score	Status	Score	Status	Score	Status
1	20.7	bad	41.14	bad	21.18	bad	40.4	bad
2	19.4	bad	36.74	bad	20	bad	33.13	bad
3	20.1	bad	39.79	bad	21.6	bad	37.35	bad
4	20.9	bad	38.61	bad	18.5	bad	32.27	bad
5	19.4	bad	36.24	bad	18.8	bad	30.94	bad
6	17.2	bad	33.15	bad	17.3	bad	27.82	bad
7	15.9	bad	35.65	bad	17	bad	28.69	bad
8	12.3	Very bad	27.70	bad	15.1	bad	25.50	bad
9	16.2	bad	33.74	bad	16.9	bad	29.17	bad
10	16.5	bad	32.55	bad	13.9	Very bad	23.59	Very bad
Average	17.86	bad	35.53	bad	18.02	bad	30.88	bad

## 4 Discussion

Rivers and running water are vital for human societies, influencing the development of cities, industrial centers, and agricultural hubs, which in turn affect water resource quality [36]. According to the research results, the Talar River has experienced severe pollution. This is evident from the data presented in Table 5, which shows that NO_3_^−^ levels are higher in summer than in winter. Specifically, the average concentration of NO_3_^−^ was 50.23 mg/L in winter and 60.21 mg/L in summer, both exceeding the WHO and EPA standards. Notably, the lowest NO_3_^−^ levels in winter were recorded at station 4, while the highest levels in summer were found at station 10. There has been a decreasing trend in NO_3_^−^ levels from the upstream station to station 4. However, from station 4 to station 10, NO_3_^−^ levels have increased. This increase is likely attributable to the proximity of the Kiakola landfill and potential leachate infiltration. The higher NO_3_⁻ concentrations in winter, compared to summer, can be explained by various factors as documented in the literature. According to the study by Li et al. (2023), seasonal variations in NO_3_^−^ sources are influenced by water flow and agricultural activities, particularly during winter and summer [37]. Also, in regions where agriculture and animal husbandry are intensive, the practice of planting and mass production can lead to excess nitrogen in the soil during winter and spring [38]. The study by Chen et al. on Taihu Lake showed that NO_3_^−^ concentrations were lower in the summer due to the dilution effect of wet deposition. In winter, sewage and manure were the primary NO_3_^−^ sources, while in summer, atmospheric deposition, along with sewage and manure inputs, played a significant role in increasing NO_3_^−^ concentrations [39]. These findings align with previous research by Qin et al. (2022) and Begum et al. (2016), who attributed elevated winter NO_3_^−^ concentrations to increased leaching from agricultural soils and runoff from upland areas, exacerbated by rainfall and snowmelt [40,41]. Conversely, Kaur's (2012) study on the Yamuna River indicated higher summer NO_3_⁻ concentrations due to increased discharge of household and industrial waste compared to winter [42]. Additionally, the average PO_4_^3−^ concentration was 0.43 mg/L in winter and 0.44 mg/L in summer, within the WHO standards. However, PO_4_^3−^ concentrations were generally higher in summer across most stations. The highest PO_4_^3−^ concentrations were observed at station 8 in both seasons, while the lowest was at station 1. A general increasing trend in PO_4_^3−^ concentration was observed from stations 1 to 8, followed by a decrease at station 10. The elevated PO_4_^3−^ levels at station 8 can be attributed to factors such as agricultural fertilizer runoff, chemical pollutants, and sewage discharge [43]. The observed increase in PO_4_^3−^ concentration is consistent with the results of the study by Roy et al. (2021) on the Shilabati River [44] and Sutamihardja et al. (2018) on the Ciliwung River [45]. However, this contradicts the findings of Kaur et al. (2012) who reported that PO_4_^3−^ concentrations increased during the monsoon season due to domestic and industrial effluents. In this study, PO_4_^3−^ levels varied between 0.029 and 0.245 mg/L in summer and 0.038 and 0.256 mg/L in monsoon [42]. The average BOD concentration was found to be 10.13 mg/L in winter and 9.48 mg/L in summer, both exceeding the WHO standard. Furthermore, these values surpass the EPA recommended limit of 3 mg/L for rivers with good water quality. Elevated BOD levels are indicative of increased organic pollution, which can lead to decreased DO levels and have detrimental effects on aquatic life [46]. The highest BOD levels were observed at station 10 in both winter and summer, indicating an upward trend. This increase is likely due to sewage inputs from Qaemshahr, Kiakola, Bahnamir, the Kiakola landfill, the Parastoo slaughterhouse, and the Sangtab industrial town. These pollutant sources have contributed to decreased DO levels and elevated BOD concentrations [47]. In studies conducted, Putri Aji et al. (2023) suggested that the elevated BOD values observed in the Ng Ringo River were likely due to domestic and industrial waste discharges, leading to a decline in water quality [48]. Also, It has been reported that higher BOD values in summer, particularly in flood seasons, are washed off by organic pollutants from nearby regions into the rivers [49,50]. On the other hand, BOD values during winter are usually low and may be due to reduced water flow and organic input during such a period, similar to the observations in the Jialing River [51] and the Nam River [52]. Furthermore, the downstream flow of the river has resulted in increased FC levels at stations 7 and 8. This trend suggests that the river traverses populated areas, receiving continuous inputs of urban, domestic, and rural sewage. Studies from various countries, including Indonesia [53], Chile [54], Bangladesh [55], and Nigeria [56], have shown that human activities, including waste from households and agricultural practices, are significant contributors to high levels of FC in rivers. Generally, higher concentrations are observed in the summer months due to increased runoff and recreational activities, while winter months often show lower levels [57]. The study by Javed et al. (2014) indicated that FC concentrations in the Kabul River were significantly higher in July (summer) compared to December (winter), primarily due to monsoon floods carrying more pollutants, thus deteriorating water quality during warmer months [58]. Among the various water quality parameters, the average EC in winter was 888.3 μS/cm, while in summer it increased slightly to 911.7 μS/cm. Both values exceeded the WHO recommended limit of 250 μS/cm. For comparison, several studies have reported EC values of rivers. For example, the Nzhelele River in South Africa had values within permissible limits, likely below 400 μS/cm, meeting WHO standards [59]. The Zab River in Asia had values ranging from 312 to 518 μS/cm, which fall within the EPA range but exceed the WHO recommendations [60]. Similarly, the central rivers of Bangladesh exhibited values between 130.8 and 366.0 μS/cm, which fall within the recommended ranges of both WHO and EPA [61]. The highest EC level was observed at station 1, with a noticeable decreasing trend in EC from upstream to downstream. However, at station 10, there is a slight increase in EC, likely due to the direct input of leachate from the Kiakola landfill. The overall decrease in EC from upstream to downstream can be attributed to various factors identified in different studies. These factors can be broadly categorized into pollution dynamics, hydrological processes, and ecosystem interactions [62]. Upstream pollution, particularly high BOD levels, decreases downstream water quality and affects EC levels. Additionally, seasonal variations, such as high temperatures, tend to exacerbate the impact of pollutants on EC levels downstream [63]. On the other hand, hydrological changes influence downstream water quality through irrigation withdrawals, land use changes, and alterations in flow patterns and sediment transport [64]. Additionally, ecosystem services and land use changes upstream can create trade-offs that affect downstream water quality. For example, agricultural practices upstream may increase nutrient runoff, leading to eutrophication downstream, which impacts both EC levels and overall water quality [65]. Regarding the Euphrates River Study, In Najaf Province, Iraq, there was no significant difference in the values of EC during winter and summer, indicating stability in ionic concentrations although other water quality parameters were worse in summer [66]. Also, a study of the Erpe River in Germany presented fluctuations in seasonal changes, mostly determined by the treated wastewater discharge, which increased in the warmer months [67]. In contrast to the findings of this study, the Batang Arau River showed an increase in EC values from upstream to downstream, reflecting the combined effects of both natural and anthropogenic activities along the river [68]. Similarly, in the detrital aquifer of Motril-Salobrena, EC values were lower near the river due to its recharge effect and increased toward the western and eastern zones, reflecting spatial variations influenced by hydrological processes [69]. The average TH was 513.6 mgCaCO_3_/L in winter and 322.5 mgCaCO_3_/L in summer. However, both values exceeded the WHO and EPA standards for water hardness in both seasons. Additionally, TH shows a downward trend from upstream to downstream, with the highest value recorded at station 1 and the lowest at station 10. Also, the TH of the river was higher in winter compared to summer. This increase in river hardness during winter can be attributed to various factors identified in different studies. For example, research on the Mahi River in Vadodara indicated a rise in TH during winter, suggesting elevated pollution levels and organic matter due to increased anthropogenic activities [70]. Similarly, studies on the Oldman River noted that calcium and magnesium levels, contributing to water hardness, increased during the fall-winter period [71]. Furthermore, investigations on the Ob River in Western Siberia revealed that the concentration of alkaline-earth metals, including calcium, decreased with discharge during winter, resulting in higher water hardness levels [72]. These findings suggest that factors like increased pollution, mineral dissolution, and seasonal variations in mineral composition contribute to the elevated TH of rivers during winter compared to summer. In this study, IRWQI_sc_ and NSFWQI were used to assess water quality. Based on the results presented in Table 6, most of the stations exhibited “bad” water quality conditions for both indicators in winter and summer. Station 8 experienced a “very bad” water quality status according to the IRWQI_sc_ in winter, while Station 10 also showed a “very bad” status for both indicators during the summer. In winter, IRWQI_sc_ scores ranged from 12.3 to 20.7, and NSFWQI scores ranged from 27.7 to 41.14. Also, In Summer, IRWQI_sc_ scores range from 13.9 to 21.6, while NSFWQI scores range from 23.59 to 40.4. As discussed, Station 8 has been identified as a major source of pollution, primarily due to the Parastoo slaughterhouse, which discharges untreated or inadequately treated sewage into the river. Conversely, at Station 10, the Kiakola landfill contributes to contamination by allowing landfill leachate to flow directly into the river, exacerbated by its low flow rate. These findings are consistent with the study by Javid et al. (2014), which concluded that the discharge of sewage from multiple factories is the primary source of pollution in the Mojen River [11]. Studies in this field have utilized various water quality indicators. For instance, a study on Chahnimeh Reservoirs in Iran revealed NSFWQI values ranging from 29.4 to 49.32, which categorized the water quality as “bad” [73]. Furthermore, a comparison between NSFWQI and IRWQI_sc_ in the Sefidroud River showed that NSFWQI identified water quality with stricter criteria, often categorizing it as “bad,” while IRWQI_sc_ provided a somewhat different assessment, indicating “relatively good” quality, The result of the index status in this study is inconsistent with the result of the present study [9]. In a study conducted by Moghadamyekta et al. (2018), the water quality status of the Godarcha watershed in Urmia was investigated using the IRWQI_sc_. The results indicated that according to the IRWQI_sc_, the tributaries and main branches of the river were classified as having water quality ranging from “relatively bad” to “relatively good” [74]. In a study on the water quality of the Songhu River in China, Zheng Li et al. (2016) noted that human activities such as industrial effluents and sewage treatment plants, as well as non-point sources like domestic sewage, livestock, and agricultural activities along with natural processes, all influence the river's water quality [75]. On the other hand, Ebrahimpour et al. (2014) assessed the water quality of Zarivar Lake in Kurdistan province using NSFWQI and found that the water quality of this lake is in the medium range [76]. The study conducted by Zohrabi et al. (2015) assessed the water quality of the Jarahi River in Khuzestan province using the NSFWQI. The results showed that during both sampling seasons, all the sampling stations were classified in the “bad” category [77]. According to the results of this study, NSWQI and IRWQI_sc_ are good indexes for evaluating the quality of water of the Talar River. These indexes can help in determining the water quality at specific stations for various purposes. In addition to the factors previously discussed, the warm climate, low rainfall, and reduced river flow during the study periods have also contributed to the water quality deterioration of the Talar River [78]. The study presented several limitations. The one-year study period may not fully capture long-term trends and seasonal variations in water quality. Additionally, the focus on a limited number of physicochemical parameters may not provide a complete picture of water quality. Moreover, the fixed sampling design may not fully represent the spatial and temporal variability of water quality. Despite these limitations, the study provides valuable insights into the current state of the Talar River and can serve as a foundation for future research and management efforts. To improve water quality in the Talar River, we recommend promoting sustainable agricultural practices such as organic methods and optimized fertilizer use to minimize nutrient runoff into the river. Additionally, enforcing stronger waste management regulations is crucial to prevent illegal dumping and reduce the impact of landfill leachate, particularly in areas like Kiakola. Establishing riparian buffer zones along the riverbanks will help filter pollutants before they enter the water, while also protecting the river's natural habitat. These measures collectively support a healthier river ecosystem and better water quality management.

## 5 Conclusion

This study reveals significant seasonal variations in water quality across the Talar River, with contaminants like COD, turbidity, and EC exceeding acceptable limits, while NO_3_^−^ and PO_4_^3−^ concentrations generally met WHO standards. Anthropogenic factors, including agricultural runoff and industrial discharges, contribute to ongoing pollution. Most sampling sites showed “bad” or “very bad” water quality, heavily influenced by sewage and landfill leachates. These findings highlight the need for comprehensive management strategies, continuous monitoring, and adaptive practices to address both point and non-point pollution sources. Future research should focus on long-term trends and the effectiveness of remediation efforts for sustainable water quality management. The outcomes of this study are crucial for the development of targeted water quality management strategies that can mitigate pollution and safeguard public health. The findings contribute to the sustainable management of water in the Talar River, which is essential for improving water availability for agriculture, ensuring safe drinking water, and protecting the ecological integrity of the region. Ultimately, this research provides a foundation for policies that will enhance water resource governance and promote long-term societal well-being.

## CRediT authorship contribution statement

**Mohammad Roshani-Sefidkouhi:** Writing – review & editing, Writing – original draft, Validation, Methodology, Investigation, Formal analysis, Data curation. **Fatemeh Mortezazadeh:** Writing – review & editing, Writing – original draft, Methodology. **Masoumeh Eslamifar:** Writing – review & editing, Methodology. **Esmaeil Babanezhad:** Writing – review & editing, Writing – original draft, Methodology, Data curation. **Masoomeh Sheikhi:** Writing – review & editing, Visualization, Methodology, Data curation. **Fathollah Gholami-Borujeni:** Writing – review & editing, Writing – original draft, Supervision, Methodology, Formal analysis, Conceptualization.

## Consent to participate

All authors have read and contributed to this manuscript and agreed to the submission.

## Consent to publish

All authors have read and contributed to this manuscript and agreed to the publication.

## Ethical approval

We confirmed that the current work is an original for the authors and has not been published or under review in any journals.

## Data availability statement

No new data was generated for the research described in the article.

## Funding

The authors did not receive support from any organization for the submitted work.

## Declaration of competing interest

The authors declare that they have no known competing financial interests or personal relationships that could have appeared to influence the work reported in this paper.
